# Antimicrobial resistance profiling of *Streptococcus suis* isolated from neurological cases in Chilean pigs

**DOI:** 10.3389/fvets.2026.1925752

**Published:** 2026-10-06

**Authors:** Fernanda Larenas-Muñoz, Francisca Videla-Martínez, Álvaro Ruiz-Garrido

**Affiliations:** Departamento de Patología y Medicina Preventiva, Facultad de Ciencias Veterinarias, Universidad de Concepción, Chillán, Chile

**Keywords:** meningitis, minimum inhibitory concentration, pigs, resistance, *Streptococcus*

## Abstract

*Streptococcus suis* is an important swine pathogen and an emerging zoonotic bacterium, with multidrug-resistant (MDR) strains representing an increasing concern for both animal and public health. This report describes the pathological findings and antimicrobial susceptibility profiles of MDR *S. suis* isolates recovered from the central nervous system of two pigs from a commercial swine farm in south-central Chile. Brain and spinal cord samples were examined by histopathology and microbiological culture, followed by bacterial identification and antimicrobial susceptibility testing using an antibiogram and minimum inhibitory concentration (MIC) analysis. Histopathological examination revealed non-suppurative inflammatory lesions characterized by mononuclear perivascular cuffing, consistent with meningoencephalomyelitis and myelitis. *S. suis* was isolated from the brain and/or spinal cord of both pigs. MIC testing demonstrated resistance to spectinomycin, ampicillin/amoxicillin, ceftiofur, penicillin, florfenicol, enrofloxacin, clindamycin, tilmicosin, tulathromycin, tylosin tartrate, tiamulin, sulfadimethoxine, trimethoprim/sulfamethoxazole, oxytetracycline, and chlortetracycline, indicating an MDR phenotype. Both isolates remained susceptible to gentamicin, while variable susceptibility was observed for danofloxacin, neomycin, tulathromycin, tiamulin, and trimethoprim/sulfamethoxazole. To the authors’ knowledge, this is the first report of MDR *S. suis* associated with neurological disease in pigs from Chile. These findings provide additional information on the antimicrobial susceptibility of *S. suis* in Chile and support the need for broader antimicrobial resistance surveillance under a One Health framework, as well as the prudent use of antimicrobials in swine production.

## 1 Introduction

*Streptococcus suis (S. suis)* is a Gram-positive coccus classified into 29 serotypes and is part of the normal microbiota of pigs (1–4). These serotypes interact with additional determinants such as sequence types (STs), virulence profiles, and phylogenetic clades, all of which influence pathogenicity and transmission dynamics both within and between animal species (3–5). This pathogen commonly colonizes the upper respiratory tract, particularly the tonsils, which represent an important reservoir for infection (6, 7). However, due to its opportunistic and zoonotic nature, under certain predisposing conditions, it can invade the bloodstream and disseminate systemically, reaching organs such as the liver, heart, spleen, and kidneys via the bloodstream (6, 7). Furthermore, *S. suis* can cross the blood–brain barrier and invade the central nervous system, where infection primarily manifests as meningitis. In addition, bloodstream invasion and systemic dissemination may lead to sepsis and other clinical manifestations, including endocarditis, arthritis, and streptococcal toxic shock syndrome (8, 9).

Globally *S. suis* infections show a heterogeneous distribution, with a marked concentration of cases in Asia, particularly those associated with serotype 2 in countries such as China and Thailand (10, 11). This pattern has been linked to cultural practices such as the consumption of raw pork and high antimicrobial pressure in pig production systems (11, 12). Nevertheless, cases have also been reported in Europe and the Americas, suggesting a global distribution of the pathogen, although infections are likely underdiagnosed (13–15).

In this context, Chile represents an epidemiological setting of particular interest. Although available evidence remains limited, the presence of *S. suis* has been confirmed in both pigs and humans, particularly among occupationally exposed workers (16–18). The limited number of reports may reflect an underestimation of its occurrence in the country, potentially associated with limitations in microbiological surveillance and the clinical similarity of *S. suis* infection to other bacterial diseases (18).

However, despite growing knowledge of the epidemiology and pathogenesis of *S. suis*, there is still a lack of integration between epidemiological, molecular, and geographic data, particularly in regions such as Latin America (13, 15, 19). This gap limits a comprehensive understanding of its transmission dynamics within a One Health framework (19, 20).

Finally, one of the most relevant features of this pathogen is its ability to acquire and disseminate antimicrobial resistance, determinants through mobile genetic elements such as integrative and conjugative elements (ICEs), which facilitate horizontal gene transfer (21–24). Antimicrobial susceptibility patterns vary among drug classes and geographically, with resistance to macrolides, oxytetracyclines and aminoglycosides differing from that reported for beta-lactams and fluoroquinolones. These mechanisms have significantly contributed to the dissemination of resistance determinants in pig production systems, with implications for both animal and human health (6, 20–26).

## 2 Materials and methods

### 2.1 Necropsy

A standardized necropsy was performed on two 10-week-old terminal hybrid pigs from an intensive swine production system located in south-central Chile. The animals originated from a PIC genetic line (337 male × 1,020 female) and exhibited neurological signs characterized by lethargy, recumbency, generalized motor weakness, forelimb paraparesis, and decreased muscle tone in the hindlimbs. Both animals were humanely euthanized due to the severity of their clinical condition and were subsequently submitted for diagnostic postmortem examination. Tissue samples, including the brain, cerebellum, spinal cord, and lung, were collected during necropsy for microbiological and histopathological analyses.

Ethical review and approval were not required because this study was based on archived tissue specimens originally collected and submitted to the Veterinary Anatomic Pathology Service as part of routine diagnostic investigations. The animals were euthanized exclusively for clinical and diagnostic purposes, independently of the present study, and all samples were collected according to standard veterinary diagnostic procedures. No animals were euthanized, subjected to additional sampling, or exposed to experimental procedures specifically for the purposes of this study.

### 2.2 Histopathological examination

For histopathological evaluation, the collected samples were fixed in 10% neutral buffered formalin routinely processed, embedded in paraffin, sectioned at 4 μm, and stained with hematoxylin and eosin (H&E), according to the standard protocols of the Histopathology Laboratory of the University of Concepción. Histological sections were examined using an AXIOSCOP 40® microscope (Carl Zeiss, Germany) equipped with an integrated AxioCam 1,200 digital camera.

### 2.3 Microbiological culture isolation and identification

Standard microbiological procedures established by the Swine Diagnostic Laboratory at the University of Concepción were followed. Prior to aseptic tissues sampling, the physical work surface was disinfected with 70% ethanol to minimize potential environmental contamination. For lung samples, the tissue surface was cauterized using a flame-heated sterile metal spatula to minimize surface contamination. An incision was subsequently made through the cauterized area using a sterile scalpel, and a sterile swab was inserted into the underlying tissue for sample collection. For brain samples, the skull was opened carefully without disrupting the meninges. Subsequently, the meninges were incised using sterile scissors and forceps, and the exposed brain surface was sampled using a sterile swab.

Each swab was sequentially inoculated onto chocolate agar, blood agar, and MacConkey agar. The primary inoculum was streaked over approximately one-quarter of the agar surface, and isolation streaking was subsequently performed using a sterile bacteriological loop. Following plate inoculation, the same swab was transferred into 5 mL of Brain Heart Infusion (BHI) broth for enrichment.

Agar plates and BHI broth cultures were incubated at 37 °C for 24 h under aerobic conditions supplemented with 5% CO₂. Following incubation, representative β-hemolytic bacterial colonies were selected from blood agar and subjected to Gram staining and catalase testing, with the latter yielding a negative result. Based on colony characteristics, including the β-hemolytic phenotype observed on blood agar, Gram-staining morphology, and the negative catalase reaction, bacterial identification was subsequently performed using the API 20 Strep biochemical identification of the isolates.

### 2.4 Determination of minimum inhibitory concentration (MIC)

Minimum inhibitory concentration (MIC) testing was performed by an external laboratory, according to its established protocols. Official breakpoints were applied according to CLSI-VET01S ED:2024 and EUCAST Clinical Breakpoints 2024 (v14.0). Where applicable, results were additionally interpreted using epidemiological cutoff values (ECOFFs), MIC50 and MIC90 values obtained from the EUCAST MIC distribution database, and data from previous studies (27–33). Quality control was performed using standard ATCC reference strains as recommended in the corresponding guidelines. For the classification of antimicrobial resistance profiles, multidrug resistance (MDR) was defined as resistance to at least three different classes of antimicrobial agents (26).

Samples were subjected to serial cultures for 72 h in liquid enrichment media at 37 °C, followed by 72 h of incubation on selective and differential solid culture media under aerobic conditions supplemented with 5% CO₂ at 37 °C. Subsequently, bacterial isolates were incubated for 18 to 25 h for MIC determination using the Sensititre® automated system. Susceptibility was evaluated against the following antimicrobials by the MIC system: Spectinomycin, Gentamicin, Neomycin, Ampicillin/Amoxicillin, Ceftiofur, Penicillin, Florfenicol, Danofloxacin, Enrofloxacin, Clindamycin, Tilmicosin, Tulathromycin, Tylosin Tartrate, Tiamulin, Sulfadimethoxine, Trimethoprim/Sulfamethoxazole, Chlortetracycline, and Oxytetracycline.

## 3 Results

### 3.1 Macroscopic findings

Both pigs had a small volume of translucent, amber-colored serous fluid within the abdominal cavity, accompanied by occasional peritoneal adhesions. Pulmonary congestion and edema were observed in both animals. In addition, pig 1 exhibited multifocal petechial hemorrhages, interpreted as postmortem changes, while pig 2 showed bronchointerstitial pneumonia involving the dorsal regions of the right middle and caudal lung lobes. Renal medullary enlargement was observed in both pigs, with bilateral involvement in pig 1 and unilateral involvement of the right kidney in pig 2. Pig 1 also had a congenital cyst within the left renal pelvis, while pig 2 exhibited a congenital renal cyst measuring 1.2 cm in diameter. In both animals, the urinary bladder was mildly distended and contained translucent yellow urine.

Gastrointestinal findings differed between the two pigs. Pig 1 exhibited diffuse duodenal hyperemia and parakeratosis of the pars esophageal, with yellowish gastric contents, whereas pig 2 had yellow mucoid gastric contents and a focal ulcer in the glandular stomach, measuring approximately 7 mm in diameter. The liver and spleen were grossly unremarkable in both animals. Mild intestinal hyperemia was observed in pig 1, while no gross intestinal abnormalities were identified in pig 2. Moderate, diffuse cerebral hyperemia was present in both pigs (Figures 1A,B), with no significant gross abnormalities identified in the cerebellum or spinal cord. Finally, subcutaneous adipose tissue was considered adequate for the age of pig 1 but was scant in pig 2.

**Figure 1:**
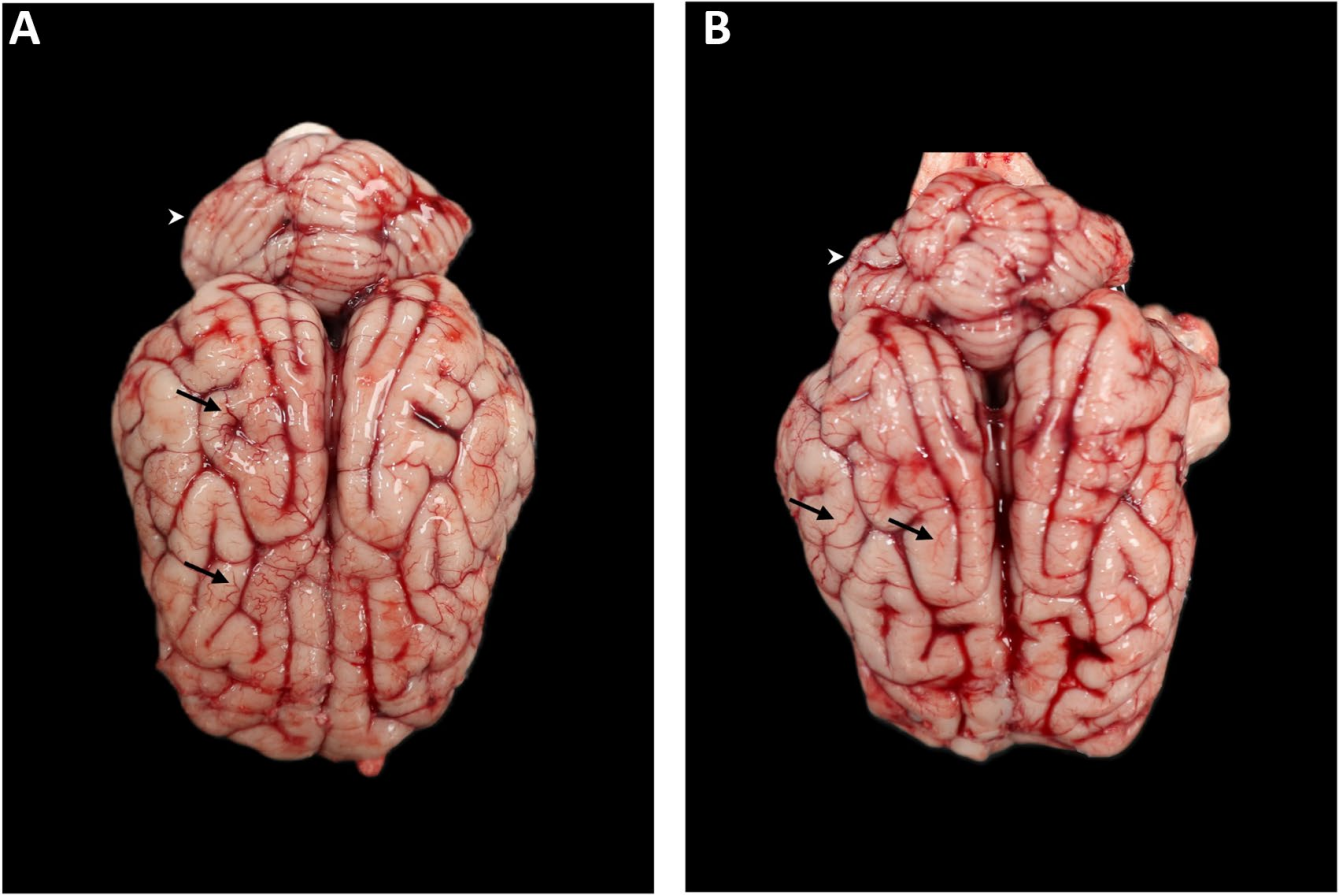
Gross appearance of the central nervous system from two pigs. **(A)** Brain and cerebellum from Pig one showing moderate diffuse cerebral hyperemia (arrows), whereas the cerebellum (arrowhead) and spinal cord showed no grossly significant macroscopic abnormalities. **(B)** Brain and cerebellum from Pig two showing moderate diffuse cerebral hyperemia (arrows), whereas the cerebellum (arrowhead) and spinal cord showed no grossly significant macroscopic abnormalities.

### 3.2 Histopathological findings

Both pigs exhibited non-suppurative inflammatory lesions within the central nervous system characterized by perivascular mononuclear cuffs. In pig 1, mild multifocal perivascular mononuclear cuffs composed predominantly of lymphocytes with occasional macrophages were observed within the gray matter of the spinal cord and the molecular layer of the cerebellum. Mild multifocal mononuclear leptomeningitis, extending into the adjacent perivascular spaces of the cerebellar cortex, was also present. No significant neuronal necrosis or suppurative inflammation was identified. These findings were consistent with mild multifocal non-suppurative meningoencephalomyelitis (see Figure 2).

**Figure 2:**
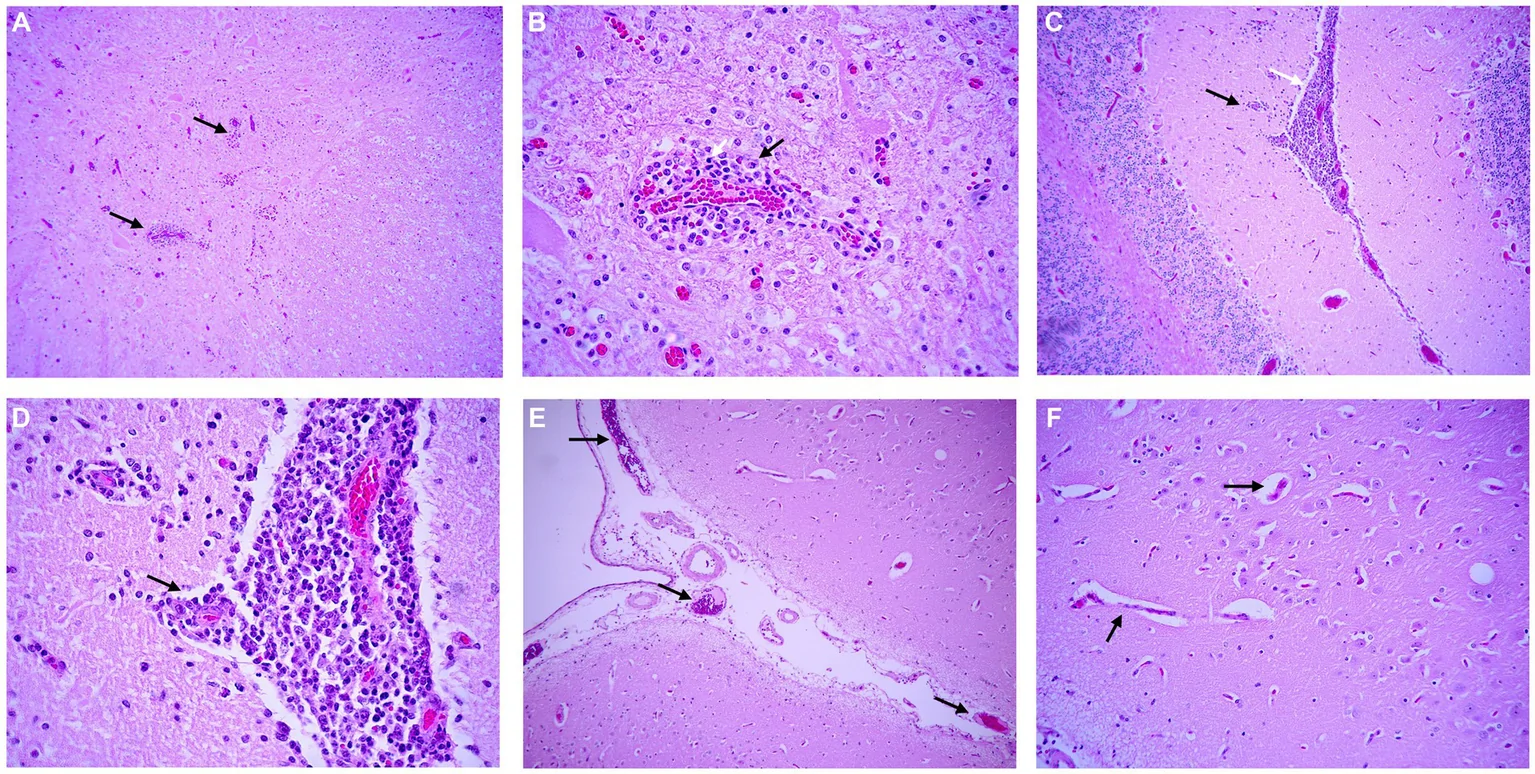
Histopathological lesions in the central nervous system of pig 1 naturally infected with *Streptococcus suis*. **(A)** Spinal cord gray matter showing mild multifocal perivascular mononuclear cuffing (arrows). **(B)** Higher magnification of the spinal cord illustrating a perivascular cuff composed predominantly of lymphocytes (white arrow) with occasional macrophages (black arrow). **(C)** Cerebellum showing mild multifocal leptomeningitis (white arrow) and perivascular mononuclear cuffing within the molecular layer (black arrow). **(D)** Higher magnification of the cerebellum illustrating moderate perivascular mononuclear cuffing extending from the leptomeninges into the adjacent cerebellar cortex (black arrow). **(E)** Cerebrum showing preserved parenchymal architecture without significant inflammatory changes and vascular congestion into meninges (arrows). **(F)** Higher magnification of the cerebrum demonstrating mild vascular congestion (arrow) with no evident neuronal degeneration or inflammatory infiltrates. Hematoxylin and eosin (H&E). Scale bars = 200 μm (insets B, D, = 50 μm and *F* = 100 μm).

In pig 2, the gray matter of the spinal cord exhibited moderate multifocal perivascular mononuclear cuffs composed predominantly of lymphocytes with occasional macrophages (Figures 3A,B). Mild vascular congestion was observed in the cerebellum (Figures 3C,D) and cerebrum without associated inflammatory infiltrates (Figures 3E,F). Overall, the lesions were consistent with moderate multifocal non-suppurative myelitis, predominantly affecting the spinal cord.

**Figure 3:**
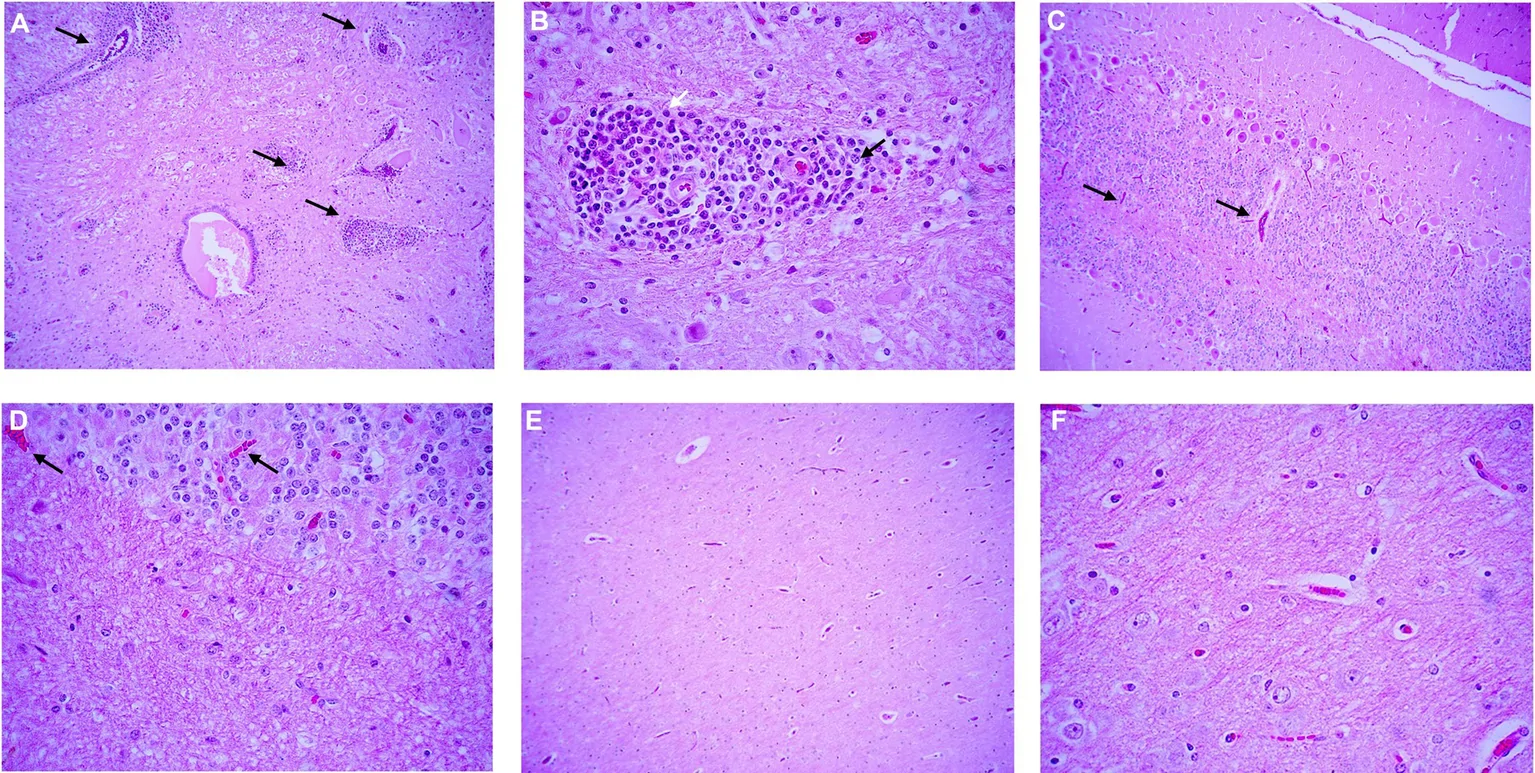
Histopathological lesions in the central nervous system of pig two naturally infected with *Streptococcus suis*. **(A)** Spinal cord showed moderate multifocal perivascular mononuclear cuffing in the gray matter (arrows). **(B)** Higher magnification showing a perivascular cuff composed predominantly of lymphocytes (white arrow) with occasional macrophages (black arrow). **(C, D)** Cerebellum with mild focal vascular congestion and preserved cortical architecture. **(E, F)** Cerebrum with mild diffuse vascular congestion and no significant inflammatory infiltrates. Hematoxylin and eosin (H&E). Scale bars = 200 μm (insets B, D and *F* = 50 μm).

### 3.3 Microbiological culture isolation and identification

*S. suis* was isolated on blood and chocolate agar and recovered from BHI broth from the brain and spinal cord of pig 1. In pig 2, *S. suis* was isolated only from the brain. In addition, *Pasteurella spp.* was isolated from the lung of pig 2.

### 3.4 Minimum inhibitory concentration

The results of the MIC testing showed that the isolates from both pigs were susceptible to gentamicin (≤1 μg/mL). Additionally, the isolate from pig 1 was susceptible to danofloxacin (0.25 μg/mL), whereas the isolate from pig 2 was susceptible to trimethoprim/sulfamethoxazole and showed intermediate susceptibility to neomycin, danofloxacin, tulathromycin, and tiamulin. Finally, both isolates were resistant to Spectinomycin, Ampicillin/Amoxicillin, Ceftiofur, Penicillin, Florfenicol, Enrofloxacin, Clindamycin, Tilmicosin, Tylosin Tartrate, Sulfadimethoxine, Oxytetracycline, and Chlortetracycline (Table 1).

**Table 1:** Minimum inhibitory concentrations (MIC, μg/mL) and antimicrobial susceptibility interpretation of *Streptococcus suis* isolates recovered from two pigs two using the SENSITITRE^®^ BOPO6F panel.

Family	Antibiotic	MIC (μg/mL)^*^	Interpretation^*^
Aminoglycosides	Spectinomycin	32/32	R/R
	Gentamicin	≤1/≤1	S/S
	Neomycin	>32/16	R/IS
Beta-lactams	Ampicillin/Amoxicillin	>16/8	R/R
	Ceftiofur	8/8	R/R
	Penicillin	8/>8	R/R
Fenicoles	Florfenicol	>8/>8	R/R
Fluoroquinolones	Danofloxacin	0.25/0.5	S/SI
Lincosamides	Clindamycin	>16/>16	R/R
	Tilmicosin	32/>64	R/R
Macrolides	Tulathromycin	32/16	R/IS
	Tylosin tartrate	32/32	R/R
Pleuromutilins	Tiamulin	>32/4	R/IS
Sulfamides	Sulfadimethoxine	>256/>256	R/R
	Trimethoprim/	>2/38/≤2/38	R/S
	Sulfamethoxazole		
Tetracyclines	Oxytetracycline	>8/>8	R/R
	Chlortetracycline	>8/8	R/R

S, sensitive; IS, intermediately sensitive; R, resistant. ^*^The left column corresponds to pig one, and the right column corresponds to pig two.

## 4 Discussion

*Streptococcus suis* has emerged as a zoonotic pathogen of global relevance, with substantial implications not only for swine production but also for human health (1, 2, 6, 8). The zoonotic risk is particularly relevant in certain occupational and cultural settings, where direct contact with pigs or their byproducts, especially in the presence of skin abrasions or wounds, represents an important route of infection. In addition, the consumption of raw or undercooked pork has been recognized as an important route of transmission (2, 8, 10, 17). The epidemiological distribution of human *S. suis* infections shows a marked concentration of cases in Asia, a pattern that has been associated, among other factors, with specific dietary practices and frequent exposure to pigs and pork products (8, 10, 11). Nevertheless, documented cases in Europe and the Americas, including human infections reported in Chile, demonstrate that *S. suis* infection is not restricted to specific endemic regions and further support its relevance as a globally distributed zoonosis (12–15, 17).

Regarding the lesions associated with *S. suis* infection in pigs, pathological findings consistent with meningitis, septicemia, arthritis, and endocarditis have been previously described (34, 35). In the present study, however, the clinical presentation was predominantly neurological, characterized by lethargy, paraparesis, and decreased muscle tone, with cerebral hyperemia observed at postmortem examination. Histopathological examination revealed cerebral hyperemia, meningoencephalomyelitis and myelitis, including nonsuppurative inflammatory changes and mononuclear perivascular cuffing. These findings differ to some extent from previous reports, in which fibrinosuppurative meningitis and myelitis have been more commonly described, whereas encephalitis and perivascular cuffing appear to be less frequently reported (34, 36). Moreover, some of the lesions reported in previous studies were described under experimental conditions or in the context of coinfections and infections caused by specific *S. suis* serotypes, which should be considered when comparing these findings with those observed in the present cases (34–39).

Antimicrobial resistance in *S. suis* should be interpreted from multiple perspectives while acknowledging the limitations of the present study. First, the inclusion of only two animals from a single farm and, consequently, only two *S. suis* isolates represents a major limitation and precludes extrapolation of the observed antimicrobial susceptibility profiles to the broader swine population in Chile. Additional limitations include the lack of molecular characterization of the isolates, particularly regarding serotyping and the detection of virulence-associated genes and antimicrobial resistance determinants.

These limitations were further compounded by resource constraints and limited access to specific diagnostic techniques, including PCR and identification by matrix-assisted laser desorption/ionization time-of-flight mass spectrometry (MALDI-TOF MS). Nevertheless, the antimicrobial susceptibility profiles observed in this study revealed resistance to multiple antimicrobial agents. Previous studies have associated similar resistance phenotypes with selective pressure, genetic diversity, and horizontal gene transfer; however, these mechanisms were not investigated in the isolates analyzed here (18–22, 40–42). Several studies have a reported a high prevalence of resistance to macrolides, sulfonamides, and tetracyclines, as well as an increasing frequency of multidrug-resistant (MDR) strains, both in sick animals and healthy carriers (23–26, 35, 40). Findings in Thailand and China show high resistance rates and the circulation of MDR strains exceeding 70%. Although direct comparison is limited by the small number of isolates examined here, both isolates showed resistance to several antimicrobial agents belonging to these classes, including tilmicosin, tylosin tartrate, sulfadimethoxine, oxytetracycline, and chlortetracycline (26, 35, 40).

One of the key factors underlying the epidemiology of antimicrobial resistance in *S. suis* is the presence of resistance determinants associated with mobile genetic elements, including transposons, plasmids, and integrative and conjugative elements (ICEs) (19, 21, 35). These elements facilitate horizontal gene transfer both among *S. suis* strains and, potentially, across bacterial species, thereby contributing to the dissemination and co-selection of antimicrobial resistance determinants (18–22). Experimental studies have shown that these elements can be transferred in bloc, generating combined resistance phenotypes, which is reflected in the frequent co-occurrence of genes such as *tet(M)*/*tet(O)* and *erm(B)*, which are involved in tetracycline and macrolide resistance (18–20, 22). Other determinants have also been described, including optrA, which is associated with resistance to oxazolidinones and phenicols in *S. suis*, further illustrating the diversity of resistance mechanisms that this species can harbor (35). Because molecular analyses were not performed in the present study, the presence of these determinants cannot be established in the two isolates. Therefore, evidence from previous studies is presented as a framework for interpreting the phenotypic resistance patterns observed here rather than as an explanation of the mechanisms involved in these isolates.

At the molecular level, resistance in *S. suis* depends not only on gene acquisition of resistance genes but also on chromosomal mutations. Alterations in penicillin-binding proteins (PBP1a, PBP2x, and PBP2b), as well as mutations affecting the quinolone target enzymes GyrA and ParC, have been associated with reduced antimicrobial susceptibility (41).

Geographical variability in antimicrobial susceptibility has also been reported for trimethoprim/sulfamethoxazole. Resistance rates range from close to 0% in some European studies to substantially higher levels in Asian reports (23–26, 40). In the present study, the isolate from pig 2 was susceptible to trimethoprim/sulfamethoxazole. However, it is important to note that this antimicrobial is used to treat respiratory and gastrointestinal infections rather than neurological infections.

A similar geographical variability has been reported for tiamulin, a pleuromutilin (23, 24). Previous studies have associated this variability with differences in antimicrobial targets and the acquisition of specific resistance determinants, whose distribution is less consistent than that of associated genes with tetracycline and macrolide resistance (21, 23, 35). However, these mechanisms were not investigated in the present isolates. This variability was also evident in the present study, as the isolate from pig 1 was resistant, while the isolate from pig 2 showed intermediate susceptibility.

It is noteworthy that both isolates showed resistance to the beta-lactams evaluated in this study. This finding contrasts with much of the available literature, in which susceptibility to beta-lactams remains common among *S. suis* populations despite the emergence of resistant isolates (23–26, 40). Beta-lactam resistance in *S. suis* has been primarily associated with the alterations in penicillin-binding proteins (PBPs), particularly PBP1a, PBP2b, and PBP2x, rather than with the acquisition of horizontally transferable resistance genes (41) However, these mechanisms were not investigated in the isolates analyzed in the present study.

Regarding aminoglycosides, both isolates were susceptible to gentamicin. As with other antimicrobials susceptibility patterns vary worldwide and may be influenced by antimicrobial use patterns in swine production (11, 24). It may also be influenced by the distribution of resistance determinants such as *aac(6′)-aph(2″)*, *aph(3′)-IIIa*, and *aadE*, compared with genes such as *tet(M)* or *optrA*, which are frequently reported in mobile genetic contexts in *S. suis* (19, 22, 26, 33, 41). However, the isolate from pig 2 showed intermediate susceptibility to tulathromycin, a macrolide. Both isolates were also resistant to spectinomycin, whereas the isolate from pig 2 showed intermediate susceptibility to neomycin. Together, these findings demonstrate phenotypic differences between the two isolates but do not allow conclusions regarding broader resistance patterns.

These differences are closely related to antimicrobial use patterns in swine production, where in Southeast Asian settings, particularly in Thailand, several studies have documented the widespread use of antibiotics for prophylactic and metaphylactic purposes, as well as the circulation of resistant and multidrug-resistant *S. suis* strains (11, 26, 40). These practices generate selective pressure on the bacterial microbiota, favoring the persistence of resistant strains (11, 24). In contrast, Europe has progressively implemented restrictive policies aimed at reducing antimicrobial use and promoting its prudent use in animal production. These measures include the prohibition of antimicrobials as growth promoters, restrictions on their prophylactic and metaphylactic use, and stricter regulation of prescription and therapeutic use. These measures include the prohibition of antimicrobials as growth promoters, restrictions on their prophylactic and metaphylactic use, and stricter regulation of prescription and therapeutic use. They are complemented by systematic surveillance programs monitoring antimicrobial consumption and bacterial resistance and by measures aimed at strengthening biosecurity, disease prevention, and livestock management practices (42, 43). This approach is also supported by recommendations from the World Health Organization (WHO) and the World Organisation for Animal Health (WOAH), as well as the European Union’s regulatory framework, within a One Health perspective (43–45). In countries with well-established antimicrobial management and surveillance programs, such as Denmark, these interventions have been associated with substantial reductions in antimicrobial consumption in animal production and improved control of antimicrobial resistance (24, 25).

In Chile, although the available evidence remains limited, the presence of *S. suis* has been documented in both swine populations and humans (14–17). The first cases of *S. suis*-associated meningitis reported in workers involved in pig production highlighted the occupational nature and zoonotic potential of this pathogen (15), while additional human cases associated with pork handling further support the relevance of exposure routes beyond direct contact with pigs on farms (17). Although antimicrobial treatment was described in these reports, national data on *S. suis* antimicrobial susceptibility profiles remain scarce. In this context, it is particularly noteworthy that porcine isolates previously reported in Chile were susceptible to penicillin (15–17), whereas both isolates analyzed in the present study were classified as resistant to this antimicrobial based on MIC results. Furthermore, the identification of *S. suis* isolates in different national studies, including genetically related strains within the areas investigated, confirms the circulation of this pathogen in Chile and underscores the need for strengthened epidemiological surveillance (14, 16).

The limited number of studies characterizing antimicrobial resistance among Chilean *S. suis* isolates makes it difficult to determine whether resistance mechanisms widely described internationally are also present and distributed among bacterial populations circulating in Chile (16, 19–22, 41, 42). Additionally, the MIC panel used in the present study did not include certain antimicrobials of interest, such as amoxicillin as an individual agent and doxycycline, thereby limiting a more comprehensive characterization of the antimicrobial susceptibility profiles. Although regulations governing antimicrobial use in animal production have advanced in recent years (43–45), a substantial gap remains in the generation of *S. suis*-specific antimicrobial resistance data. This lack of information limits comparisons with other regions and hinders assessment of temporal trends in resistance and their potential relationship with implemented control measures. Therefore, although international evidence indicates that *S. suis* can harbor a diverse range of antimicrobial resistance determinants and associated mobile genetic elements (19–22, 41, 42), their presence, distribution, and epidemiological relevance among isolates circulating in Chile remain to be determined through dedicated phenotypic and molecular studies.

Another noteworthy finding was that neither isolate was susceptible to all antimicrobial agents evaluated. Although the limited number of isolates precludes broader conclusions, this observation is consistent with previous reports describing the widespread occurrence of antimicrobial resistance among *S. suis* populations (23–27, 40, 42). Beyond its role as an important swine pathogen, *S. suis* has also been recognized as a potential reservoir of antimicrobial resistance determinants, some of which are associated with mobile genetic elements and may contribute to horizontal gene transfer between bacterial populations (18–22, 41). These characteristics further underscore the relevance of *S. suis* antimicrobial resistance from a *One Health* perspective (18, 42–46). Based on their phenotypic susceptibility profiles, the two isolates analyzed here exhibited resistance to multiple antimicrobial classes and would meet previously proposed definitions of multidrug resistance (47). While the lack of comprehensive genetic characterization represents a significant limitation, our findings, along with the available evidence, support the view that antimicrobial resistance in *S. suis* is a complex and dynamic evolutionary phenomenon shaped by the selective pressure imposed by antimicrobial use (11, 24, 43–45). Chile is not exempt from this global challenge (14–17); however, the scarcity of national studies addressing *S. suis* and its antimicrobial susceptibility profiles limits our understanding of the extent and distribution of antimicrobial resistance in Chilean swine populations. This represents a significant knowledge gap and highlights the need for more epidemiological, phenotypic, and molecular studies at the national level.

## Data Availability

The original contributions presented in the study are included in the article/Supplementary material, further inquiries can be directed to the corresponding author/s.
